# Successful Treatment of Severe Laryngomalacia Due to Posterior Collapse of the Epiglottis by Correction of Glosso-Larynx (CGL): A Case Report

**DOI:** 10.3390/children13020223

**Published:** 2026-02-05

**Authors:** Toshiro Yamanishi

**Affiliations:** Yamanishi ENT Clinic, 518 Waseda Tsurumakicho, Shinjuku-ku, Tokyo 162-0041, Japan; yamany915@yahoo.co.jp

**Keywords:** laryngomalacia, infant airway obstruction, correction of glosso-larynx (CGL), pediatric otolaryngology, minimally invasive surgery, tongue–larynx dynamics, sleep-disordered breathing, upper airway management

## Abstract

**Highlights:**

**What are the main findings?**

- Correction of glosso-larynx (CGL) effectively improved airway stability and respiratory symptoms in an infant with severe laryngomalacia.
- The procedure enhanced feeding ability and overall clinical condition without perioperative complications.

**What are the implications of the main findings?**

- Addressing tongue–larynx dynamics may provide a novel therapeutic option for selected cases of severe laryngomalacia.
- CGL may offer an alternative minimally invasive approach in the management of pediatric upper airway obstruction.

**Abstract:**

Background: Laryngomalacia is the most common cause of inspiratory stridor in infancy. While most mild cases resolve spontaneously, severe cases may require surgical intervention. We report a case of severe laryngomalacia successfully treated with correction of glosso-larynx (CGL), a surgical procedure originally developed for ankyloglossia with deviation of the epiglottis and larynx (ADEL). Methods: A 2-month-old infant with severe symptoms was evaluated using objective anatomical and functional metrics. The patient underwent CGL under local anesthesia to release restrictive tension on the hyoid-larynx complex. Results: The patient showed rapid and sustained improvement in respiratory symptoms. At the one-month follow-up, endoscopic examination confirmed functional airway expansion with limited to no morbidity. Conclusions: This case demonstrates that CGL may represent a feasible and minimally invasive therapeutic option for selected cases of severe laryngomalacia, particularly those involving complex tongue–larynx dynamics.

## 1. Introduction

Laryngomalacia is the most frequent cause of inspiratory stridor during infancy. Due to the fragility of the supraglottic structures, inspiratory collapse or narrowing of the larynx may occur, leading to cyanosis, feeding difficulty, poor weight gain, respiratory distress, and obstructive sleep apnea [1,2].

In mild cases, spontaneous improvement is expected, and careful observation is usually sufficient. However, in severe cases, surgical intervention may be required [3,4,5]. Conventional surgical treatments include supraglottoplasty using laser or microdebrider techniques, epiglottopexy, and tracheostomy. Although these procedures can be effective, each is associated with specific disadvantages, and a safer, less invasive, and more definitive treatment approach is desirable.

Supraglottoplasty involves resection or contraction of the epiglottis, aryepiglottic folds, or arytenoid mucosa using a CO_2_ laser or microdebrider to enlarge the airway. While this approach is minimally invasive and can be performed endoscopically, postoperative restenosis, aspiration due to excessive resection, and supraglottic stenosis remain concerns [6,7,8,9].

Epiglottopexy stabilizes the epiglottis by anterior or superior fixation to prevent airway collapse and can provide immediate airway improvement. However, the risk of swallowing dysfunction and the potential need for reoperation with growth are recognized limitations [10,11,12,13,14]. A serious complication common to these procedures is supraglottic stenosis or edema, which may ultimately necessitate tracheostomy in severe cases. Although tracheostomy provides reliable airway control, it significantly impairs long-term quality of life and is therefore reserved for the most severe, treatment-refractory cases [11,12,13,14].

Mukai et al. reported that surgical treatment for ankyloglossia with deviation of the epiglottis and larynx (ADEL) using correction of glosso-larynx (CGL) improves respiratory function by anterior traction of the larynx, preventing supraglottic collapse, with favorable outcomes and minimal complications [15,16] (Table 1). This procedure involves the intentional incision of the genioglossus muscle bundle, which modulates the position of the hyoid bone and tongue base to expand the pharyngeal airway. Specifically, CGL is a distinctive approach involving a deep, 10–15 mm incision into the genioglossus muscle bundle to release the abnormal tension pulling the larynx forward. It is a simple, minimally invasive technique that does not require an external cervical incision.

However, clinical experience with this procedure remains limited, and further accumulation of cases is needed to establish its efficacy and safety. Here, we report a case of severe laryngomalacia successfully treated with CGL.

## 2. Case Presentation

### 2.1. Patient Profile

A 2-month-old female infant was referred to our department with a constellation of symptoms including snoring, obstructive sleep apnea (OSA), difficulty initiating and maintaining sleep, feeding difficulties, poor weight gain, persistent coughing, and cyanosis during crying.

### 2.2. Perinatal and Family History

The patient was born at 38 weeks and 2 days of gestation via normal vaginal delivery in the cephalic position. The duration of labor was 2 h and 22 min, and birth weight was 2878 g. There were no remarkable prenatal or perinatal complications. She was the second child; the first child was healthy. The family history was significant for hereditary upper airway obstruction: both the paternal grandmother and the biological father suffered from OSA, requiring Continuous Positive Airway Pressure (CPAP) therapy.

Despite the severity of symptoms, no pharmacological interventions, such as acid suppressants or nasal steroids, were prescribed prior to the initial consultation at our department.

From the first day of life, frequent regurgitation and poor facial coloration were noted. Although discharged on day 5, she subsequently developed abdominal distension and sleep apnea. At 25 days of age, she was evaluated at an emergency department for poor feeding and marked abdominal distension; however, she was diagnosed only with constipation. By one month of age, the patient developed labored breathing during sleep, feeding difficulties, and frequent choking. Additionally, livedo reticularis and recurrent maternal breast complications were noted. During a routine pediatric check-up, the only finding addressed was poor weight gain, and a policy of expectant management was recommended regarding the respiratory symptoms.

Despite this, the mother remained deeply concerned due to several clinical signs, including frequent detachment from the nipple during feeding, excessive aerophagia, perioral pallor, and cyanosis during crying. Other notable physical findings included frontal skin darkening and prominent wrinkling during crying, erect hair, and periorbital tension. Although the parents reconsulted their primary pediatrician due to persistent respiratory distress—characterized by difficulty falling asleep, shallow sleep, snoring, and obstructive apnea—no respiratory diagnosis was made, and no medications were prescribed. Throughout this period, the mother suffered from frequent mammary complications, including mastitis and milk stasis (nipple white spots), necessitating bi-weekly visits to a midwife for breast care. The parents gradually developed insomnia due to chronic anxiety over the patient’s abnormal breath sounds and apneic episodes present since birth. Upon consulting the midwife, a potential otorhinolaryngological etiology was suggested. Consequently, the patient was referred to our department and underwent an initial evaluation on postnatal day 59.

### 2.3. Initial Examination

**Figure 1:**
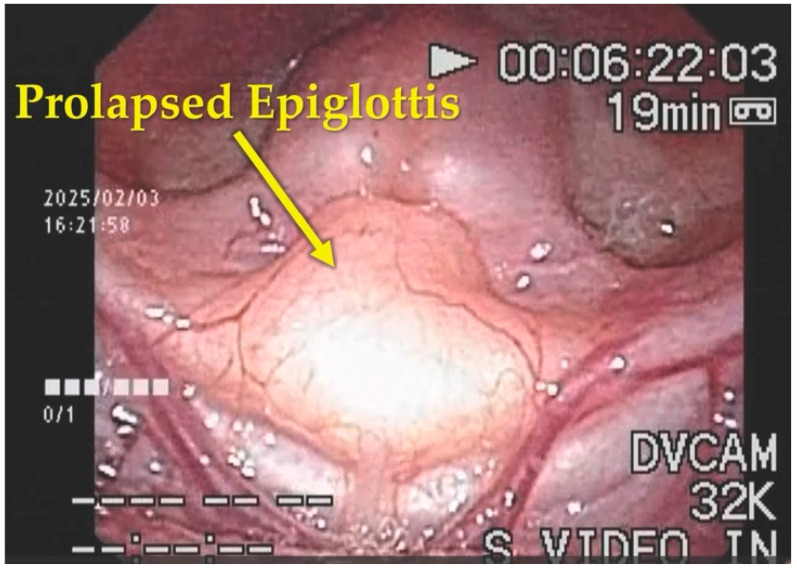
Flexible endoscopic findings of the larynx at initial presentation. Initial laryngoscopy during inspiration revealed marked posterior and inferior prolapse of the epiglottis, leading to significant narrowing of the glottic opening. These findings are consistent with Olney classification type 3 laryngomalacia (see also Supplementary Video S1).

At presentation, poor facial coloration and inspiratory stridor during crying were observed. Oxygen saturation during feeding ranged from 93% to 98% (baseline), decreasing to 91% during crying. Flexible laryngoscopy revealed inspiratory collapse of the epiglottis into the glottis, causing airway obstruction, consistent with Olney classification type 3 laryngomalacia [13] (Figure 1) (Supplementary Video S1).

**Figure 2:**
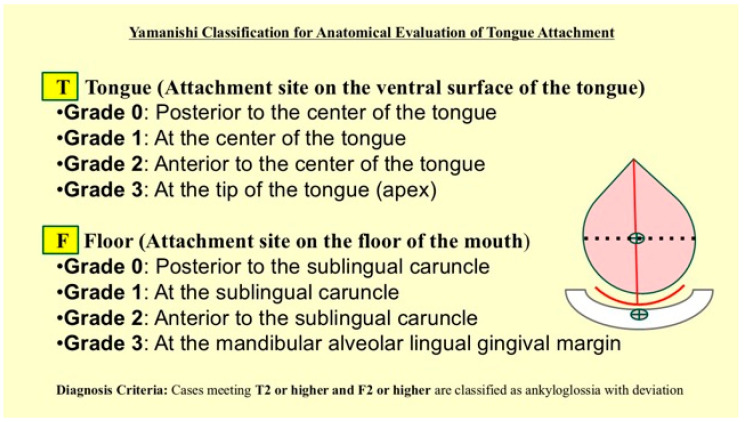
Yamanishi Classification for Anatomical Evaluation of Tongue Attachment.

**Figure 3:**
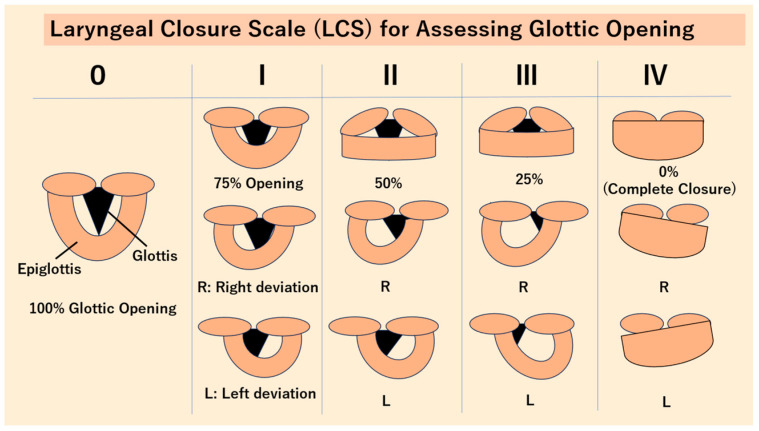
Laryngeal Closure Scale (LCS) for Assessing Glottic Open. 0: The glottis is 100% open (completely open). I: The glottis is 75% open. II: The glottis is 50% open. III: The glottis is 25% open. IV: The glottis is not visible at all (complete closure). R: The larynx is deviated to the right. L: The larynx is deviated to the left. Note: The Yamanishi Classification and LCS were previously presented at the 34th and 35th Annual Meeting of the Japan Society for Ankyloglossia with Deviation of the Epiglottis and Larynx (2024–2025) and published in the *Orthodontics Yearbook 2025* (Quintessence Publishing Co., Ltd., Tokyo, Japan).

The patient exhibited hypoxemia, necessitating urgent intervention. Given the clinical presentation, immediate surgical intervention was deemed essential. The surgical indication was established based on objective anatomical and functional evaluations. Anatomical severity was assessed using the Yamanishi Classification, which categorizes the tongue’s attachment and posterior displacement [17] (Figure 2). The patient was identified as Class T3/F3, strongly suggesting a definitive diagnosis of severe ADEL (Ankyloglossia with Deviation of the Epiglottis and Larynx). This pathology exerts intense restrictive tension on the hyoid-larynx complex, leading to a high risk of laryngeal collapse. Additionally, the Laryngeal Closure Scale (LCS) was utilized via flexible laryngoscopy to quantify the functional degree of inspiratory obstruction (Figure 3). The patient was graded as LCS Grade IV (complete closure), which mandated immediate surgical intervention. Laryngopharyngeal fiberscopy revealed no signs of acid reflux, such as redness of the arytenoid region or pooling of saliva in the hypopharynx, which are characteristic of laryngopharyngeal reflux disease. Given the severity of the laryngomalacia, it was determined that conservative management with gastric acid suppressants would be insufficient to address the immediate clinical risk.

The parents expressed profound relief upon the confirmation of a definitive diagnosis, yet they remained visibly exhausted and deeply anxious regarding the patient’s clinical course to date. While they fully comprehended the potential for grave complications associated with severe laryngomalacia, they strongly requested a minimally invasive intervention to achieve immediate symptomatic improvement. Notably, they had been informed by a referring midwife that CGL could serve as an effective treatment for ADEL to improve laryngomalacia. Consequently, while conventional options such as epiglottopexy remained standard considerations, we proposed the surgical approach for ADEL as described by Mukai et al. Following a comprehensive explanation of the procedure, its physiological rationale, and anticipated outcomes, the parents provided informed consent, and we proceeded with the surgery.

### 2.4. Surgical Procedure

**Figure 4:**
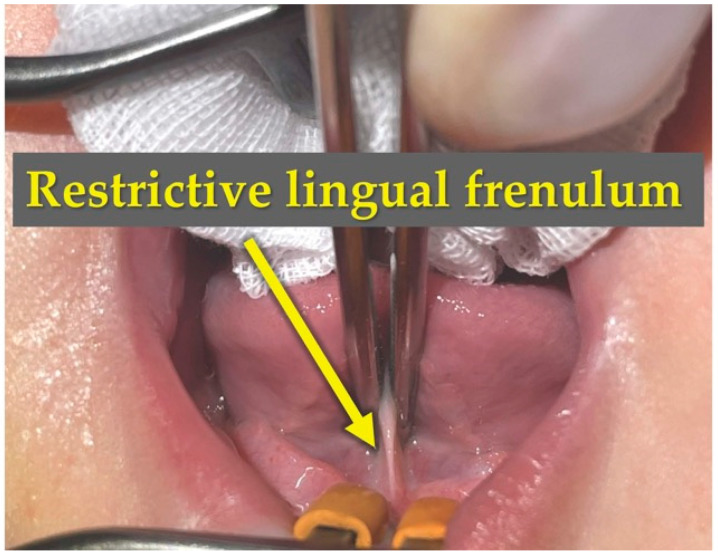
Intraoperative findings: Identification and exposure of the lingual frenulum. Under infiltration anesthesia, the patient was placed in the supine position. The surgical field was prepared to identify the restrictive lingual frenulum. The lingual frenulum was first incised to expose the underlying genioglossus muscle, with the laser positioned for the procedure.

**Figure 5:**
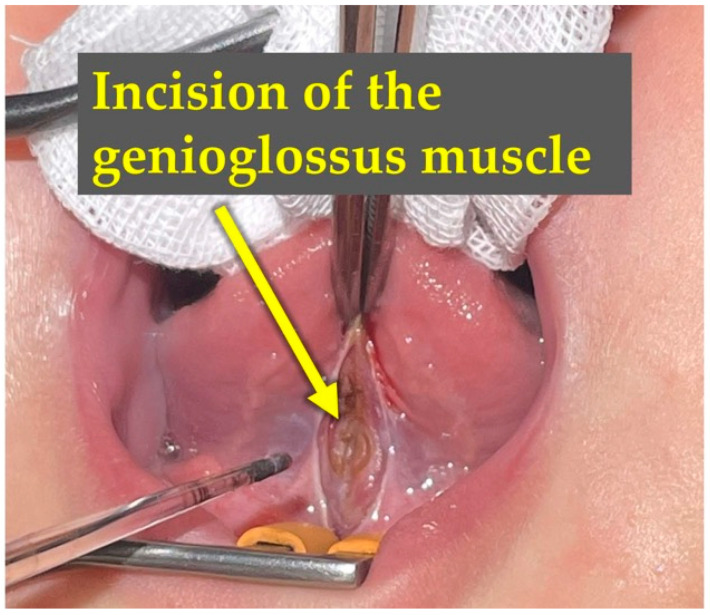
Surgical release: Incision of the deeper genioglossus muscle bundle during the CGL procedure. Following the mucosal incision, the deeper fibers of the genioglossus muscle were carefully identified and incised. This maneuver aims to release the restrictive tension on the hyoid-larynx complex. No significant bleeding was observed, demonstrating the minimally invasive nature of the procedure.

CGL was performed on postnatal day 60 (the day after the initial consultation). The patient was placed in the supine position, and Xylocaine jelly (1 mg) was applied to the floor of the mouth for infiltration anesthesia. The lingual frenulum was incised using a diode laser, followed by exposure of the genioglossus muscle. A single layer of the genioglossus muscle bundle was incised (Figure 4 and Figure 5). The incision was made to a depth of 10–15 mm to sufficiently release the hyoid-laryngeal complex. The specific intraoperative technique, including the laser incision and the management of the genioglossus muscle, is demonstrated in Supplementary Video S2.

### 2.5. Postoperative Course

Immediately after the surgery, inspiratory stridor was relieved; however, due to transient agitation caused by the procedure, intense crying and transient hypopnea were observed. The patient was placed in a sitting position with continuous SpO_2_ monitoring, which fluctuated between 91% and 100%. Oxygen was administered via mask at 3 L/min and discontinued after 3 min once SpO_2_ stabilized at 100%.

Although inspiratory stridor was absent, SpO_2_ variability persisted, and parental concern led to referral and admission to another pediatric hospital for observation. During hospitalization, conservative management for gastroesophageal reflux was provided, including feeding posture guidance, administration of mosapride and rikkunshito, right lateral positioning after feeding, and enemas. Upper airway obstruction symptoms were not prominent. The patient was discharged after a 4-day hospitalization for postoperative observation.

**Figure 6:**
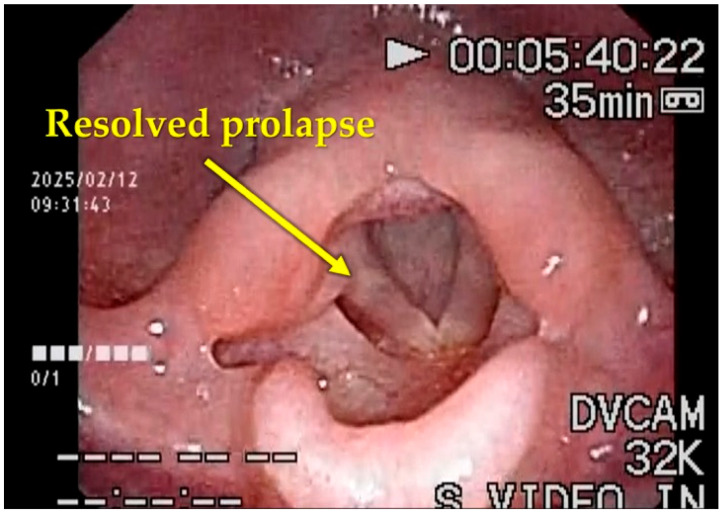
Flexible endoscopic laryngeal findings after CGL surgery. Follow-up endoscopy on postoperative day 8 demonstrated a dramatic improvement in laryngeal stability. The previous inspiratory collapse of the epiglottis was completely resolved, and the pharyngolaryngeal airway was significantly expanded (see also Supplementary Video S3).

At postoperative day 8, follow-up examination showed marked improvement. Facial coloration was normal, feeding difficulties resolved, cyanosis during crying disappeared, and SpO_2_ remained stable at 97–100%. Flexible laryngoscopy demonstrated dramatic improvement, with complete resolution of epiglottic prolapse during inspiration (Figure 6) (Supplementary Video S3). Findings remained stable at one-month follow-up.

## 3. Discussion

Laryngomalacia is the most common cause of inspiratory stridor in infancy. Approximately 90% of mild cases resolve spontaneously by one year of age [18,19,20]. However, severe cases may present with feeding difficulty, failure to thrive, respiratory distress, and obstructive sleep apnea, necessitating active intervention [3].

Since the 1980s, aggressive surgical management has been adopted in Western countries for severe laryngomalacia [19,21,22]. Supraglottoplasty is commonly used for Olney type 1 and 2, while epiglottopexy is typically reserved for type 3 cases. Although favorable outcomes have been reported, complications such as aspiration, bleeding, granulation, and supraglottic stenosis occur in approximately 4% of cases and may require tracheostomy [3,7,23].

It should be emphasized that while supraglottoplasty remains the primary standard of care for severe laryngomalacia, CGL may be considered a potentially safe and effective alternative or adjunct in carefully selected patients—particularly those with anatomical features of ADEL—rather than a replacement for established surgical approaches.

The clinical efficacy of CGL in this case is primarily attributed to the release of the restrictive tension exerted by the genioglossus muscle bundle on the hyoid-larynx complex. Unlike conventional lingual frenotomy, the targeted incision of the deeper genioglossus fibers alleviates the pathological backward pull, thereby allowing the hyoid bone to relocate to a more anatomically favorable anterior-inferior position [16,24]. This structural realignment releases the extrinsic compression on the supraglottic space and facilitates a definitive functional expansion of the pharyngolaryngeal airway. By prioritizing the release of abnormal muscular dynamics over simple mucosal resection, CGL achieves sustainable stabilization of the laryngeal inlet.

This report has limitations, including the single-case design and limited follow-up duration. Furthermore, it should be acknowledged that acid reflux treatment was initiated for one week following the patient’s transfer to another pediatric hospital postoperatively. However, the improvement in SpO_2_ levels was already documented upon admission to that hospital, prior to the clinical onset of the medication’s effect. This chronological sequence strongly suggests that the surgical intervention was the primary driver of the rapid clinical improvement, although the concomitant reflux therapy may have provided supportive benefits.

Optimal timing of intervention and long-term outcomes require further investigation. Although some cases of laryngomalacia may improve spontaneously [18,19,20], the early months of life are critical for auditory, speech, and neurodevelopmental milestones. In this critical period, impairment of the three primary functions of the larynx—namely, respiration, lower airway protection, and phonation—must be avoided. Hypoxia during this period confers no developmental benefit. Also, recent evidence indicates that acid suppression provides little or no benefit in infants with laryngomalacia [25]. This strongly suggests that surgical intervention is the primary driver of clinical improvement. Procedures such as CGL, which can safely alleviate airway obstruction in severe cases, may therefore be considered as a feasible early intervention option for selected cases involving complex tongue–larynx dynamics.

## 4. Conclusions

Correction of glosso-larynx (CGL) was effective in treating severe laryngomalacia in this infant, with rapid improvement and no major complications at the one-month follow-up. Accumulation of further cases is necessary to establish the safety and efficacy of this minimally invasive procedure.

## Figures and Tables

**Table 1:** Comparison of CGL and conventional surgical procedures.

Feature	Conventional Procedures	CGL (Present Method)
Surgical Approach	Endolaryngeal	Transoral
Incision Details	Excision of laryngeal tissue	Genioglossus myotomy (10–15 mm)
Operation Time	Several hours	A few minutes
Anesthesia	General anesthesia	Topical/infiltration anesthesia
Hospitalization	Required	Not required
Tracheostomy	May be required	Not required (avoided)
Recovery	Gradual (weeks to months)	Immediate improvement
Primary Risk	Granuloma, stenosis	Small sample size

## Data Availability

No new data were created or analyzed in this study.

## Supplementary Materials

The following supporting information can be downloaded at: https://www.mdpi.com/article/10.3390/children13020223/s1, Supplementary Video S1: Flexible endoscopic laryngeal findings at the initial presentation. Supplementary Video S2: Intraoperative findings of the CGL surgery. Supplementary Video S3: Flexible endoscopic laryngeal findings after surgery.
