# Correction: Yamanishi, T. Successful Treatment of Severe Laryngomalacia Due to Posterior Collapse of the Epiglottis by Correction of Glosso-Larynx (CGL): A Case Report. *Children* 2026, *13*, 223

**DOI:** 10.3390/children13040555

**Published:** 2026-04-16

**Authors:** Toshiro Yamanishi

**Affiliations:** Yamanishi ENT Clinic, 518 Waseda Tsurumakicho, Shinjuku-ku, Tokyo 162-0041, Japan; yamany915@yahoo.co.jp

## Missing Acknowledgement

In the original publication [1], Acknowledgement section was not mentioned. The Acknowledgements have now been inserted and should read:

“The author wishes to express his deepest gratitude to his late mentor, Susumu Mukai, who first introduced the concept of ADEL and provided invaluable surgical guidance. This work is respectfully dedicated to his memory. The author serves as the President of the Japan ADEL Society and gratefully acknowledges the Society’s academic collaboration and continued support in advancing the clinical and scientific understanding of ADEL. The author is profoundly indebted to Katsutoshi Oda, Department of Obstetrics and Gynecology, The University of Tokyo, for his insightful advice, thoughtful discussions, and generous academic support. Furthermore, the author extends his sincere appreciation to Shigeo Akira, Director of Meirikai Tokyo Yamato Hospital, for his leadership in establishing the Institutional Review Board and for facilitating the ethical review process essential to the preparation of this clinical report. Finally, the author expresses his heartfelt gratitude to the patient and the patient’s family for their trust, understanding, and kind cooperation, which made this clinical report possible.”

## Text Correction

A correction has been made to Institutional Review Board Statement.

The corrected statement appears below:

“The study was conducted in accordance with the Declaration of Helsinki and approved by the Ethics Committee of Meirikai Tokyo Yamato Hospital (Approval No. 23, 15 May 2025). This work was carried out with the academic cooperation of the Japan ADEL Society, of which the author, Toshiro Yamanishi, serves as President.”

The authors state that the scientific conclusions are unaffected. This correction was approved by the Academic Editor. The original publication has also been updated.

## References

[1] Yamanishi T. (2026). Successful Treatment of Severe Laryngomalacia Due to Posterior Collapse of the Epiglottis by Correction of Glosso-Larynx (CGL): A Case Report. Children.

